# Overexpression of the Ets-1 transcription factor in human breast cancer

**DOI:** 10.1038/sj.bjc.6602128

**Published:** 2004-09-07

**Authors:** Y Buggy, T M Maguire, G McGreal, E McDermott, A D K Hill, N O'Higgins, M J Duffy

**Affiliations:** 1 Department of Surgery, University College Dublin, St Vincent's University Hospital, Dublin 4, Ireland; 2 Conway Institute of Biomolecular and Biomedical Research, University College Dublin, Dublin 4, Ireland; 3 Department of Nuclear Medicine, St Vincent's University Hospital, Dublin 4, Ireland

**Keywords:** Ets transcription factors, HER-2/*neu*, urokinase plasminogen activator (uPA), invasion, breast cancer

## Abstract

The Ets family of transcription factors regulate expression of multiple genes involved in tumour progression. The aim of this study was to investigate the expression of Ets-1 in a large panel of human breast cancers and relate its levels to the parameters of tumour progression and metastasis. Using RT–PCR, Ets-1 mRNA was detected in 30 out of 42 (71%) fibroadenomas and 131 out of 179 (73%) primary breast carcinomas. Similarly, levels of Ets-1 mRNA were not significantly different in fibroadenomas and primary breast carcinomas. Using Western blotting, four forms of the Ets-1 protein were detected, that is, p33, p42, p51 and p52. Levels of both p51 and p52 but not p33 and p42 were present at significantly higher levels in the carcinomas compared to the fibroadenomas (for p51, *P*<0.007; for p52, *P*<0.02; Mann–Whitney *U*-test). Levels of p52, p51 and p33 correlated significantly with uPA protein levels (*P*<0.01), while only levels of p52 correlated significantly with HER-2/*neu* protein levels (*P*<0.01). Using immunohistochemistry, Ets-1 was found predominantly in tumour cells, but was also detected in some stromal cells surrounding tumour islands. We conclude that, while at the mRNA level, Ets-1 was found at similar levels in fibroadenomas and primary breast carcinomas, higher protein levels were detected in the cancers compared to the benign specimens. Since p52, p51 and p33 correlate with uPA levels, these forms of Ets-1 may play a role in breast cancer metastasis.

E26 transformation-specific (*Ets*) genes encode a family of transcription factors, which have been shown to regulate a diverse array of biological functions (Wasylyk *et al*, 1993). The criterion for membership of this family is an approximate 85-amino-acid consensus motif known as the Ets domain (Papas *et al*, 1989; Janknecht and Nordheim, 1993; Wasylyk *et al*, 1993; Mavrothalassitis *et al*, 1994; Tymms and Kola, 1994). The Ets motif is necessary for the specific recognition of a purine-rich core sequence, GGAA/T, within the promoter/enhancer regions of multiple target genes (Higashino *et al*, 1995; Himelstein *et al*, 1997). This domain appears to possess a unique structural motif for binding DNA with high specificity (Wasylyk *et al*, 1993). Amongst the genes whose promoter/enhancer regions contain Ets-binding sites (EBSs) are several matrix-degrading proteases such as the urokinase plasminogen activator (uPA), cathespin B, matrix metalloproteinase (MMP)-1, MMP-3 and MMP-9 (White *et al*, 1997; Kitange *et al*, 1999; Sementchenko and Watson, 2000; Behrens *et al*, 2001) and genes coding for adhesion proteins such as specific integrins, cadherins and selectins (Sementchenko and Watson, 2000).

Ets-1, the founder member of the *Ets* gene family, is located on the long arm of chromosome 11 (11q23–q24). Ets-1 is the cellular homologue of the *v-Ets* gene found in the avian erythroblastosis virus E26 (Watson *et al*, 1990). In humans, the *Ets-1* gene encodes two mRNA transcripts, a full-length (6.8 kb) transcript and an alternatively spliced (2.7 kb) transcript (Koizumi *et al*, 1990; Collyn d'Hooghe *et al*, 1993). The full-length transcript encodes both a 51 kDa protein and a 42 kDa protein (Bhat *et al*, 1996). The 51 kDa protein can be phosphorylated as a result of increased intracellular calcium levels, giving rise to a 52 kDa protein (Bhat *et al*, 1996). The 42 kDa protein lacks exon VII, which contains the major phosphorylation site in the full-length protein (Jorcyk *et al*, 1991).

Ets proteins are targets for phosphorylation in response to stimulation by a variety of different growth modulators, including intracellular calcium, activators of protein kinase C pathways, growth factors and cytokines (Bhat *et al*, 1996). Variations in the degree of activation of the Ets-1 protein seen in different cell types may arise from differential phosphorylation or dephosphorylation within a given cell (Pognonec *et al*, 1990).

In model systems, increased expression of Ets-1 was found to be associated with enhanced angiogenesis and the invasive phenotype (Vandenbunder *et al*, 1994a, 1994b; Ito *et al*, 1998; Sato *et al*, 2000). In addition, several angiogenic factors, such as the vascular endothelial growth factor (VEGF) and both acidic and basic fibroblast growth factor (FGF), have been shown to induce expression of Ets-1 (Wernert *et al*, 1994; Sato *et al*, 2000). In a preliminary report, Span *et al* (2002) recently showed that high expression of Ets-1 was associated with adverse prognosis in breast cancer. In that study, the authors also found a significant correlation between Ets-1 levels and both VEGF and plasminogen activator inhibitor-1 (PAI-1) (Span *et al*, 2002). All these findings, taken together, suggest that Ets-1 is likely to play a key role in cancer progression, especially angiogenesis and invasion.

In this study, we investigated the *ex vivo* expression of Ets-1 in a panel of human breast tissues using reverse transcriptase–polymerase chain reaction (RT–PCR), Western blotting and immunohistochemistry. We also related levels of Ets-1 to the established prognostic factors for breast cancer and factors involved in tumour progression, that is, urokinase plasminogen activator (uPA) and human epidermal growth factor receptor 2 (HER-2/*neu*).

## MATERIALS AND METHODS

### Human breast tissues

Breast tissue specimens were obtained during surgery, and, following histopathological examination, the remainder of each sample was immediately frozen in liquid nitrogen. The samples were then powdered using the Braun Mikrodismembrator (Braun Apparate, Melsungen, Germany) and the powder stored at −80°C until further use. The characteristics of the breast cancers used in this study are summarised in Table 1

**Table 1:** Characteristics of breast cancers used

**Variable**	***n***	**% positive**
*Nodal status*		
Negative	80	44.6
Positive	89	49.7
Unknown	10	5.7

*Tumour size*		
T1 (⩽2 cm)	45	25
T2 (2–5 cm)	91	51
T3–4 (⩾5 cm)	29	16
Unknown	14	8

*Histology type*		
Ductal (D)	133	74.3
Lobular (L)	27	15
D & L	10	5.7
Unknown	9	5

*ER status*^a^		
Negative	50	28
Positive	128	71.5
Unknown	1	0.5

*PR status*^b^		
Negative	73	41
Positive	77	43
Unknown	29	16

ER=oestrogen receptor. PR=progesterone receptor.

a Cutoff point=200 fmol g^−1^ of wet weight tissue.

b Cutoff point=1000 fmol g ^−1^ of wet weight tissue.

. In total, 179 primary breast carcinomas were analysed for Ets-1 mRNA expression and of these 78 were randomly selected for analysis of Ets-1 protein expression. In all, 42 fibroadenomas were analysed for Ets-1 mRNA expression, 38 of which were tested for Ets-1 protein expression. Immunohistochemistry was carried out on cryosections from seven primary breast carcinomas and seven fibroadenomas. The quality of each section and the relative cellular composition were determined by histopathological assessment. Ethical approval for this study was issued by the Ethics Committee, St Vincent's University Hospital, Dublin, Ireland.

### RNA extraction

Total RNA was extracted from 100–200 mg of breast tissue using the guanidine isothiocyanate/phenol chloroform method (Chomczynski and Sacchi, 1987). The integrity of the RNA was visualised by running 10 *μ*l on a 2.5% agarose gel with ethidium bromide staining and checking the integrity of the 28 S and 18 S bands. The purity of RNA was determined by reading the absorbance at 260 nm.

### Complementary strand DNA (cDNA) synthesis

Total RNA (1 *μ*g) was reversed transcribed to single-stranded cDNA in a final volume of 20 *μ*l. The reaction mixture contained 0.4 mM of each deoxynucleotide triphosphates (dNTP), 10 *μ*g ml^−1^ of Oligo (dT)_12–18_, 10 mM dithiolthreitol (DTT), 50 mM Tris–HCl (pH 8.3), 75 mM KCL and 3 mM MgCl_2_. This reaction mixture was incubated for 5 min at 70°C to remove secondary RNA structures, centrifuged and cooled on ice, followed by the addition of 4.6 U of human placenta ribonuclease inhibitor (GibcoBRL®) and 200 U of Moloney murine leukaemia virus reverse transcriptase (Promega). Samples were incubated for 1 h at 37°C and finally heated for 5 min at 65°C. cDNA was stored aliquoted at −20°C until required for PCR amplification.

### PCR

Semiquantitation of Ets-1 and uPA was performed following co-amplification and normalisation with an internal control sequence, glyceraldehydes-3-phosphate-dehydrogenase (GAPDH). The primers for Ets-1 and GAPDH were designed and numbered according to Genbank notation (Accession numbers=NM_005238, NM_002046). Primers specific for the amplification of the transcripts were chosen using the ‘Primer Select’ software, and the specificity confirmed by carrying out a detailed BLAST search. Details of all primer pairs are as follows:

*Ets-1:* sense, 5′-CGC TAT ACC TCG GAT TAC TT-3′; antisense, 5′-GTC ATA GGA GGG AAC ACG-3′ (nucleotides 735–754 and 1113–1130, respectively).

*GAPDH*: sense, 5′-CCA CCC ATG GCA AAA TTC CAT GGC A-3′; antisense, 5′-TCT AGA CGG CAG GTC AGG TCC ACC-3′ (nucleotides 227–250 and 801–824, respectively; Crofford *et al*, 1993).

The PCR reaction comprised of 2 *μ*l of cDNA template obtained from 1 *μ*g of RNA, 100 ng each of upstream and downstream primers (Genosys, Pampisford, UK), 0.25 mM each of dNTP, 2.0 mM MgCl_2_ and 1.25 U of Taq DNA polymerase (Promega) in a reaction buffer made up to a final volume of 50 *μ*l. All PCR reactions were performed in an automated thermocycler (MJ Research, Watertown, MA, USA). The cycling conditions for each of the primer sets were as follows:

*Ets-1:* A denaturing step for 2 min at 94°C, followed by 1 min at 94°C, 1 min at 58°C and 1 min at 72°C for 33 cycles, followed by 5 min at 72°C.

*GAPDH:* A denaturing step for 2 min at 94°C, followed by 1 min at 94°C, 1 min at 60°C and 1 min at 72°C for 30 cycles, followed by 5 min at 72°C.

With these conditions, amplification products were obtained in the exponential phase for both sets of primers used. Following amplification, 20 *μ*l of PCR product from each reaction was run out on a 2.5% agarose gel and visualised by ethidium bromide staining under UV light. The intensity of the bands was determined by densitometry (EagleEye™, Stratagene, UK), and expressed as a ratio of the GAPDH band intensity, which was used as an internal control. Negative controls included omission of reverse transcriptase and replacement of cDNA by water. The identity of the PCR products was confirmed by direct sequencing (ABI prism 310 technology). For all reactions, the product agreed with the expected sequence ⩾98%.

### Western blot analysis

Frozen powdered tissue samples were suspended in 50 mM Tris–HCl (pH 7.4) (2 ml per 100 mg of sample) containing Triton X-100 to a final concentration of 1% (v v^−1^). These homogenates were agitated for 20 min at 4°C and centrifuged at 13 000 **g** for 20 min at 4°C. The pellet was discarded and the supernatant containing the protein was transferred to a clean tube. Total protein concentration was determined using the micro-bicinchoninic acid (BCA) protein assay (Pierce, Rochford, IL, USA) according to the manufacturer's instructions. Samples each containing 30 *μ*g of protein, along with molecular weight marker (SeeBlue ™ Pre-stained standards, Novex, USA) and a positive control for the antibody (Jurkat cell lysate, Upstate Biotechnology, Lake Placid, NY, USA), were subjected to 12% polyacrylamide gel electrophoresis under reducing conditions, and the proteins were transferred to a nitrocellulose membrane (Sigma Chemical Company, St Louis, MO, USA).

After nonspecific sites were blocked with 5% powdered milk in 0.05% Triton X-100/Tris-buffered saline (TBS-T) for 1 h, blots were incubated overnight with an IgG-purified rabbit polyclonal Ets-1 (C-20) antibody at a final concentration of 0.4 *μ*g ml^−1^ (Santa Cruz Biotechnology, Inc) in a solution containing 5% powdered milk and 0.05% Triton X-100/TBS. The blots were then washed three times in TBS-T for 10 min each and incubated with a HRP-conjugated goat anti-rabbit IgG (Sigma-Aldrich Ireland Ltd, Dublin, Ireland) at a concentration of 1 *μ*g ml^−1^ in 5% powdered milk in 0.05% TBS-T. All samples were also blotted for *β*-actin (Clone AC-15, Sigma-Aldrich, Ireland Ltd, Dublin, Ireland) to normalise protein amounts. Bands were detected by the addition of a chemiluminescent substrate (Luminol system, Santa Cruz Biotechnology, Inc). Blots were exposed to Fuji X-ray film for 3 min. Scanning densitometry was performed on the protein bands using the EagleEye™ Still Video system (EagleEye™, Stratagene, UK) and arbitrary units assigned. Values are expressed as a ratio to *β*-actin.

### Immunohistochemistry

Breast tissue was placed directly in the cryopreservative embedding media OCT compound (Tissue Tek, Sakura, Finetek, Europe BV, Zoeterwoude, The Netherlands) and immediately frozen in liquid nitrogen. Sections (7 *μ*m) were placed on glass slides coated with 2% 3-amino-propyl-triethoxy-silane (Sigma-Aldrich Ireland Ltd, Dublin, Ireland) in acetone and dried for 12 h at room temperature. Tissue sections were fixed in 1% paraformaldehyde, air-dried and incubated for 15 min at room temperature with normal blocking serum (Vectastain Goat Elite Kit, Vector Laboratories Ltd, Peterborough, UK). The primary antibody for Ets-1 (C-20) was an IgG-purified rabbit polyclonal antibody raised against the peptide mapping at the carboxy-terminus of human Ets-1 (200 *μ*g ml^−1^; Santa Cruz Biotechnology, Inc). The primary antibody was diluted 1 : 100 in 0.6 M NaCl. Following 1 h incubation of the primary antibody at room temperature, a biotinylated rabbit secondary antibody (1 : 500) (Vector Laboratories, Burlingame, CA, USA) was applied to the sections, followed by the avidin–biotin–peroxidase complex (ABC kit, Vectastain, Burlingame, CA, USA). Negative controls included preabsorption of the primary antibody with an excess of corresponding synthetic peptide (sc-350 p, 100 *μ*g 0.5^−1^ ml, Santa Cruz Biotechnology, Inc), and the use of isotype-matched nonimmune rabbit IgG.

### Immunofluorescent microscopy

Immunofluorescent microscopy was carried out using the same procedure as that used for immunohistochemistry. Sections were incubated in diluted normal goat serum (Vector Laboratories, Burlingame, CA, USA). The primary polyclonal antibody for Ets-1 (C-20), as described previously, was diluted 1 : 10 in 10% normal human serum and incubated for 60 min. Sections were washed in phosphate-buffered saline (PBS) and incubated for 30 min in biotinylated anti-rabbit secondary antibody (1 : 500, Vector Laboratories, Burlingame, CA, USA). Slides were washed thoroughly in PBS, and incubated in a 1 : 100 dilution of Cy3 fluorochrome-conjugated monoclonal mouse anti-biotin antibody (BN-34, Sigma Chemical Company, St Louis, MO, USA) for 30 min. This was added to bind the biotinylated anti-rabbit secondary antibody. Following washing with PBS, the slides were mounted in Dako® fluorescent mounting medium (Dako Corporation, Carpinteria, CA, USA). Negative controls included the use of an isotype-matched nonimmune rabbit IgG and omission of the primary antibody.

### Enzyme-linked immunosorbent assay (ELISA)

uPA ELISA kits were obtained from American Diagnostica Inc, Greenwich, CT, USA; oestrogen receptor (ER) and progesterone receptor (PR) ELISA kits were obtained from Abbott Diagnostics, North Chicago, IL, USA, and the HER-2/*neu* ELISA kits were obtained from Oncogene Research products, Cambridge, MA, USA. The supernatants prepared for Western blotting were used in the ELISA assays. All procedures were carried out using the manufacturers' recommended protocol. The cutoff values for ER and PR were 200, and 1000 fmol G^−1^ wet weight tissue, respectively (Cullen *et al*, 2001).

### Statistical analysis

The strength of associations between the various parameters measured in this study was tested using nonparametric tests, which do not assume that the data are normally distributed. Mann–Whitney *U*-tests were used for categorical data, and the Spearman rank correlation was used for continuous variables. Two-sided *P*-values below 0.05 were considered statistically significant.

## RESULTS

### Expression of Ets-1 mRNA in fibroadenomas and primary breast carcinomas

**Figure 1:**
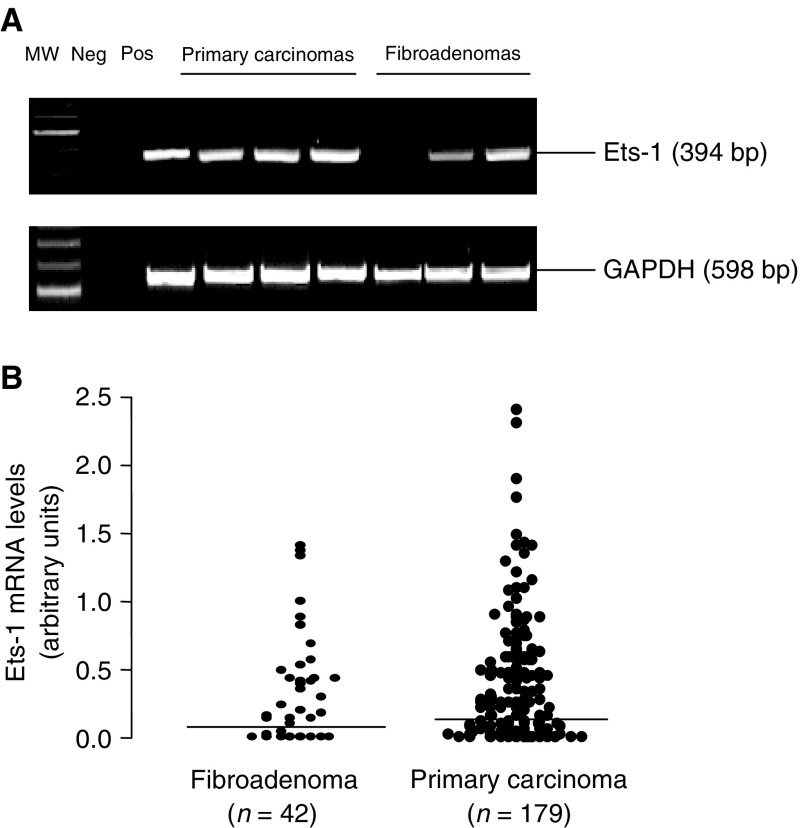
(**A**) Representative RT–PCR products of Ets-1 and GAPDH detected in primary carcinomas and fibroadenomas; lane MW, 100 base pair DNA ladder; lane 1, negative control without RT; lane 2, positive control for RT–PCR. (**B**) Distribution of Ets-1 mRNA levels in fibroadenoma tissue and primary breast carcinomas as detected by RT–PCR. Median levels are indicated by the bars. Each dot represents a semiquantitative value following scanning densitometry from one experiment. Values are expressed as a ratio to GAPDH.

Representative expressions of Ets-1 and GAPDH mRNA detected by RT–PCR are shown in Figure 1A

. GAPDH was used as an internal control and confirmed an equivalent amount of RNA loading for each sample used. Ets-1 mRNA was expressed in similar proportions of fibroadenomas (30 out of 42, 71%) and primary breast carcinomas (131 out of 179, 73%) (*P*, NS). Although levels tended to be higher in the carcinomas compared to the fibroadenomas, this difference was not statistically significant (Figure 1A, B).

### Expression of Ets-1 protein in fibroadenomas and primary breast carcinomas

Figure 2A

**Figure 2:**
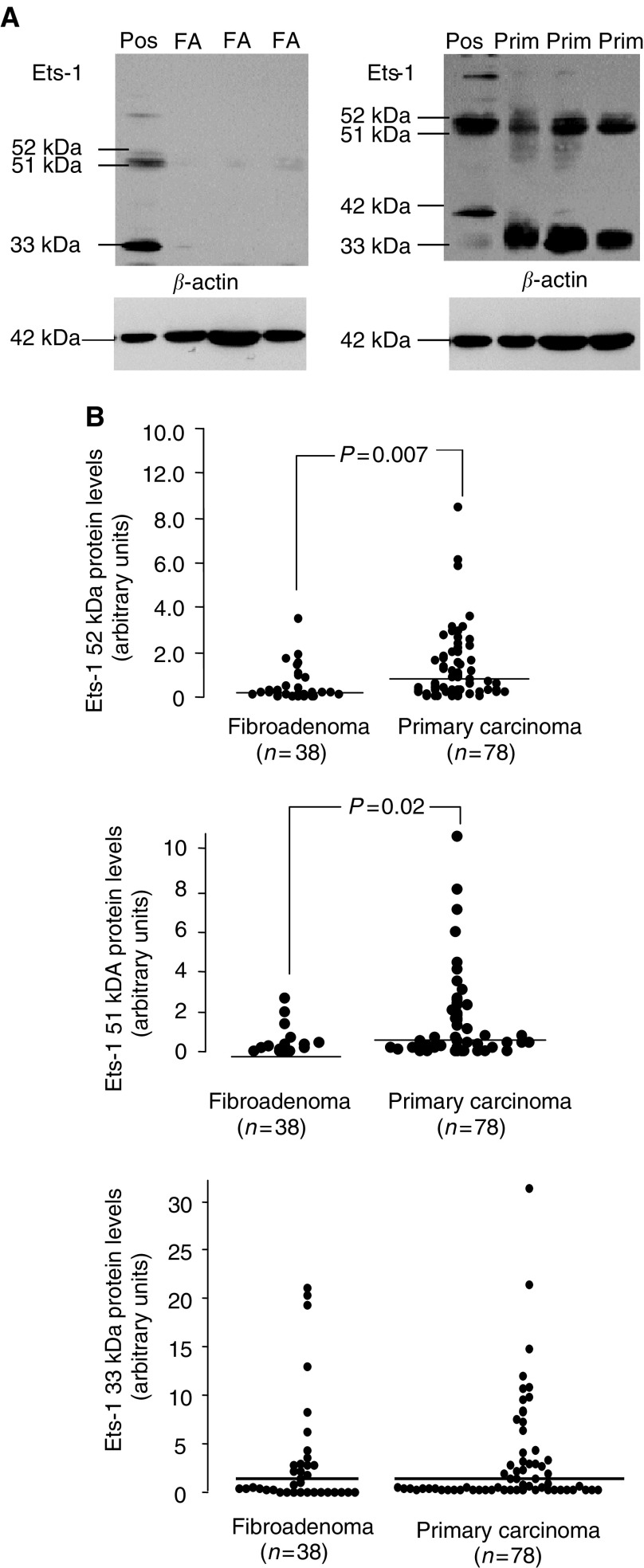
(**A**) Ets-1 protein expression in primary carcinomas and fibroadenomas. Levels of Ets-1 protein were determined by Western blotting as described under ‘Materials and methods’. FA denotes fibroadenoma tissue, Prim. denotes primary breast carcinomas and Pos. denotes positive control (Jurkat cell lysate). Arrows show Ets-1 p51, p52, p33 and p42 proteins detected using an Ets-1-specific antibody. (**B**) Distribution of Ets-1 p52, p51 and p33 levels in primary carcinomas and fibroadenomas as detected by Western blotting. Median levels are indicated by the bars. Each dot represents a semiquantitative value following scanning densitometry from one experiment. Values are expressed as a ratio to *β*-actin.

shows Ets-1 protein expression measured by Western blotting. In total, four different proteins were detected: p51, which is regarded as full-length Ets-1, p52, which is thought to be a phosphorylated form of Ets-1 (Koizumi *et al*, 1990), p42, which is derived from a splice variant lacking exon VII (Bhat *et al*, 1996), and p33. The p33 form of Ets-1 does not appear to have been described previously in the literature. However, as this band was eliminated in the presence of excess blocking peptide (sc-350 p, 100 *μ*g 0.5^−1^ ml, Santa Cruz Biotechnology, Inc), (data not shown), it is likely to be a form of Ets-1 or at least Ets-1 related. Immunoreactivity of p42, p51 and p52 was also eliminated in the presence of excess blocking peptide (data not shown).

Table 2

**Table 2:** Distribution of the main forms of the Ets-1 protein in fibroadenomas and primary breast carcinomas

	**Fibroadenoma (*n*=38)**			**Primary breast carcinoma (*n*=78)**		
	**No. pos.**	**% pos**	**Median level**	**No. pos.**	**% pos**	**Median level**
p33	27	74	0.44	52	67	0.36
p42	5	13	0	8	10	0
p51	18	47	0	44	56	0.19
p52	32	84	0.18	65	83	0.44

summarises the distribution of the four forms of the Ets-1 protein in fibroadenomas and primary breast carcinomas. For both carcinomas and fibroadenomas, the predominant form was p52, followed by p33 and p51. In the carcinomas, p42 was detected in only eight out of 78 (10%) samples, while in the fibroadenomas this form was present in five out of 38 (13%) samples. Levels of both p51 and p52, but not p33, were significantly higher in carcinomas compared to fibroadenomas (for p51, *P*=0.007; for p52, *P*=0.02; Mann–Whitney *U*-test).

**Figure 3:**
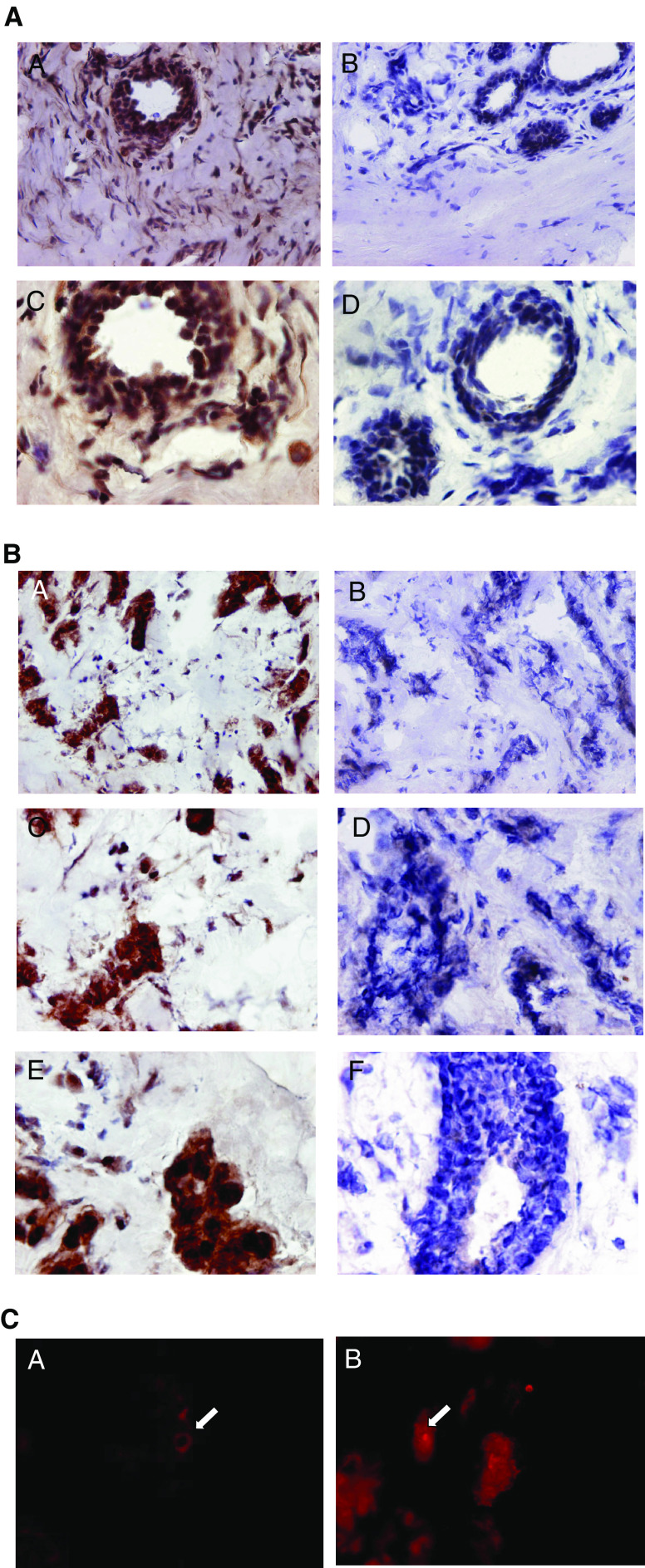
(**A**) Ets-1 staining of frozen sections of fibroadenoma. Immunohistochemical staining of fibroadenoma tissue showing Ets-1 protein distributution mainly around the lobules and ducts and also in the stromal fibroblasts. Some negatively stained cells are also seen in the surrounding stromal tissue. Immunoperoxidase staining of sections was carried out using the Vectastain Elite ABC kit (Vector Laboratories) on 7 *μ*m sections of frozen breast tissue embedded in OCT according to the manufacturer's recommendations. (A) Fibroadenoma tissue section × 20 (objective); (B) rabbit IgG-negative control × 20 (objective); (C) × 40 showing Ets-1 staining in the epithelial cells lining the ducts and also in the surrounding stroma; (D) IgG control × 40. (**B**) Ets-1 staining of frozen sections of breast tumour tissue. Immunohistochemical staining of breast tumour tissue showing Ets-1 protein distributution mainly in the tumour cells and to a lesser extent in the stromal fibroblasts surrounding the tumour islands. Some negatively stained cells are also seen in the surrounding stromal tissue. Immunoperoxidase staining of sections was carried out using the Vectastain Elite ABC kit (Vector Laboratories) on 7 *μ*m sections of frozen breast tissue embedded in OCT according to the manufacturer's recommendations. (A) Breast tumour tissue section × 20 (objective); (B) rabbit IgG negative control × 20 (objective); (C) × 40 showing Ets-1 staining in the tumour cells; (D) IgG control × 40; (E) × 60 showing a cluster of tumour cells stained positively for Ets-1; (F) IgG control × 60. (**C**) Localisation of Ets-1 protein in breast tumour tissue by immunofluorescent microscopy. Ets-1 staining is observed in both the nucleus and cytoplasm of the tumour cells in the primary breast cancer tissue sections. (A) Arrow shows Ets-1 cytoplasmic staining (original magnification × 200); (B) arrow shows Ets-1 nuclear staining.

Typical immunostaining patterns for Ets-1 in primary breast carcinoma tissue and fibroadenoma tissue sections are shown in Figure 3

. Haematoxylin and eosin-stained sections were used to select the most cellular part of the tissue sections and the area with the lowest architectural differentiation. We found strong Ets-1 staining in the primary breast carcinoma tissue sections (Figure 3A, B), especially on the tumour cells (Figure 3B). Some stromal cell staining was also observed, especially surrounding the tumour islands. Most of the Ets-1 staining was found to be nuclear, although some cytoplasmic staining was also seen. In the fibroadenomas, epithelial and stromal cell staining was also observed (Figure 3). The specificity of Ets-1 antibody staining was confirmed by an absence of reactivity on serial sections treated with Ets-1 antibody that had been preincubated with an excess of specific antigen (result not shown). Immunostaining with isotype-matched nonimmune rabbit IgG yielded a complete absence of staining in all sections stained (result not shown). Immunofluorescent microscopy was also carried out on the primary breast carcinoma sections (*n*=7). Both nuclear and cytoplasmic localisations of Ets-1 were observed (Figure 3C).

### Relationship between Ets-1 protein and uPA

**Figure 4:**
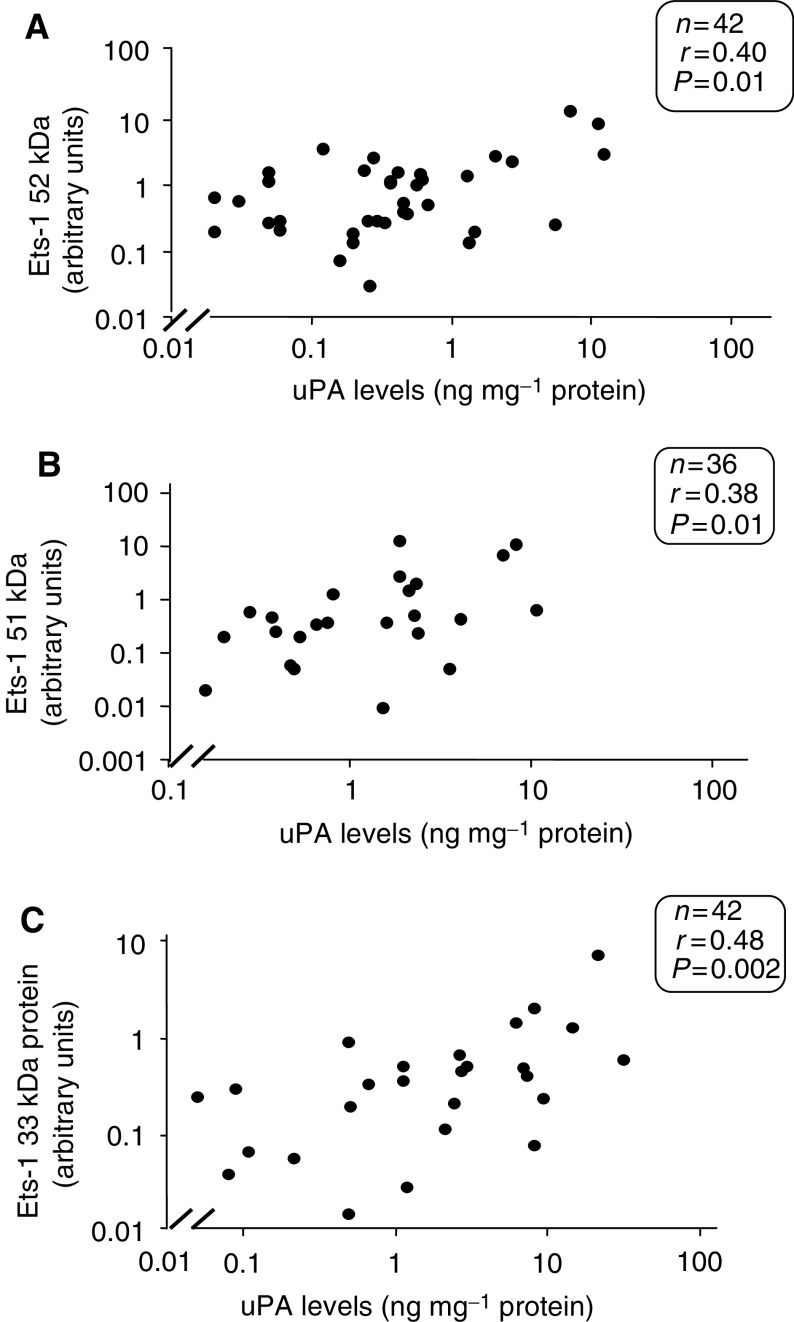
Relationship between Ets-1 52 kDa and uPA protein (**A**) and Ets-1 51 kDa protein and uPA (**B**) and Ets-1 33 kDa and uPA (**C**). Ets-1 protein measurements were carried out using Western blotting. Arbitrary units were assigned to each protein band following scanning densitometry. Values are expressed as a ratio to *β*-actin. uPA protein was measured using ELISA. Data was analysed using the nonparametric Spearman rank test.

Since uPA is causally involved in tumour progression (Duffy *et al*, 1988), we correlated its levels with those of Ets-1. As shown in Figure 4

, a statistically significant relationship was found to exist between uPA and the 52, 51 and 33 kDa forms of the Ets-1 protein in the primary carcinomas (for p51, *r*=0.38, *P*=0.01; for p52, *r*=0.40, *P*=0.01; for p33, *r*=0.48, *P*=0.002; Spearman rank nonparametric test) (Figure 4A–C).

### Relationship between Ets-1 and established prognostic factors in breast cancer

Neither Ets-1 mRNA nor any form of the Ets-1 protein correlated significantly with tumour size, nodal status, histology type ER or PR levels (Table 3

**Table 3:** Ets-1 mRNA and protein expression in different subsets of primary breast carcinomas

	**Ets-1 mRNA**		**Ets-1 p52**		**Ets-1 p51**		**Ets-1 p42**		**Ets-1 p33**	
	***n***	**No. Pos (%)**	***n***	**No. Pos. (%)**	***n***	**No. Pos. (%)**	***n***	**No. Pos. (%)**	***n***	**No. Pos. (%)**
*Nodal status*										
Negative	80	62 (78)	32	27 (84)	32	18 (56)	32	6 (19)	32	22 (69)
Positive	89	63 (71)	39	33 (85)	39	22 (56)	39	1 (2.5)	39	26 (67)
Unknown	10		7		7		7		7	

*Tumour size*										
T1 (⩽2 cm)	45	34 (76)	17	15 (88)	17	14 (82)	17	1 (6)	17	15 (88)
T2 (2–5 cm)	91	68 (75)	38	30 (79)	38	16 (42)	38	4 (11)	38	21 (55)
T3–4 (⩾5 cm)	29	18 (62)	18	15 (83)	18	10 (56)	18	1 (6)	18	11 (61)
Unknown	14		5		5		5		5	

*Histology type*										
Ductal (D)	133	98 (70)	61	53 (87)	61	31 (51)	61	6 (9.8)	61	40 (66)
Lobular (L)	27	20 (74)	7	6 (86)	7	6 (86)	7	1 (14)	7	5 (71)
D & L	10	7 (70)	6	2 (33)	6	4 (67)	6	0 (0)	6	4 (44)
Unknown	9		4		4		4		4	

*ER status*^a^										
Negative	50	37 (74)	20	15 (75)	20	10 (50)	20	1 (5)	20	13 (65)
Positive	128	93 (73)	56	49 (88)	56	33 (59)	56	6 (11)	56	37 (66)
Unknown	1		2		2		2		2	

*PR status*^b^										
Negative	73	53 (73)	36	30 (83)	36	16 (44)	36	3 (8)	36	26 (72)
Positive	77	93 (73)	35	30 (86)	35	23 (66)	35	3 (9)	35	20 (57)
Unknown	29		7		7		7		7	

**Figure 5:**
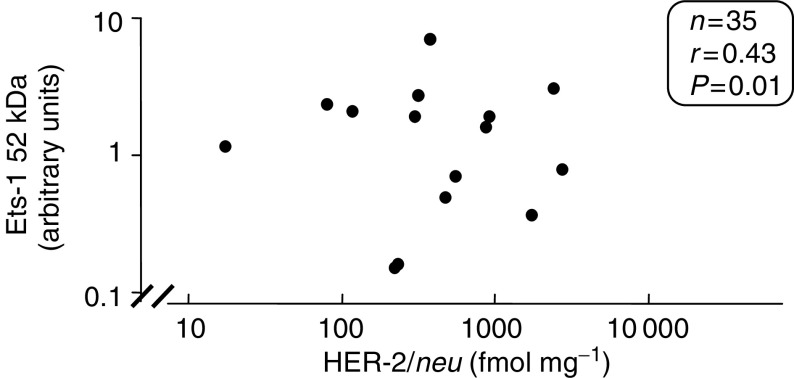
Relationship between Ets-1 52 kDa protein and HER-2/*neu* (fmol mg^−1^). Ets-1 protein measurements were carried out using Western blotting. Arbitrary units were assigned to each protein band following scanning densitometry. Values are expressed as a ratio to *β*-actin. HER-2/*neu* levels were measured using ELISA. Data was analysed using the nonparametric Spearman rank test.

ER=oestrogen receptor. PR=progesterone receptor.

a Cutoff point=200 fmol g^−1^ of wet weight tissue.

b Cutoff point=1000 fmol g^−1^ of wet weight tissue.

). Although there were relatively few matched values for Ets-1 protein and HER-2/*neu* (*n*=35), a significant relationship was observed between the phosphorylated form of the Ets-1 protein (52 kDa) and HER-2/*neu* levels (*r*=0.43, *P*=0.01; Spearman rank nonparametric test) (Figure 5

). No significant relationship however was found between p51, p33, p42 and HER-2/*neu* levels.

## DISCUSSION

Ets proteins regulate the expression of multiple genes involved in cancer dissemination and have been associated with invasiveness in model systems (Sementchenko and Watson, 2000). Despite this, a distinct biological role for the Ets proteins in human cancer has yet to be established. In this investigation, Ets-1 mRNA was found to be expressed at similar levels in breast carcinomas and fibroadenomas. In contrast to findings at the mRNA level, concentrations of the two main protein forms of Ets-1, that is, p51 and p52, were significantly increased in carcinomas compared to the fibroadenomas. The increase in expression of Ets-1 protein compared to Ets-1 mRNA in the primary carcinomas may relate to enhanced translation in the malignant *vs* the benign tissues. Previously, using *in situ* hybridisation, mRNA for Ets-1 was reported to be increased in invasive breast cancers *vis-à-vis* in *in situ* lesions (Behrens *et al*, 2001). To our knowledge, no previous report has compared Ets-1 expression in fibroadenomas and carcinomas.

In addition to p51 and p52, two further forms of the Ets-1 protein, that is, p42 and p33, were detected in the present investigation. p42 is derived from an mRNA splice variant lacking exon VII and has previously been detected in both benign and malignant breast tumours (Behrens *et al*, 2001). In MDA-MB-231 breast cancer cells but not MCF-7 cells, overexpression of the p42 form of Ets-1 was found to reduce cell survival (Ballschmieter *et al*, 2003). On the other hand, in colon cancer cells, the p42 form of the Ets-1 protein was shown to rescue Fas-induced apoptosis (Li *et al*, 1999). The p33 protein band reported here does not appear to have been described previously, and its identity remains unknown. A p39 form of Ets-1 previously reported by Koizumi *et al* (1990) was not detected in this study. This form of the Ets-1 protein is thought to be derived from the p51 protein by covalent modification of the protein by a synthetic protease inhibitor and thus may be a product of the specific extraction method (Fisher *et al*, 1992).

In this investigation, we found the Ets-1 protein to be present in both tumour and stromal cells, especially when the latter surrounded tumour islands. Using both immunohistochemistry and *in situ* hybridisation, Behrens *et al* (2001) detected expression of Ets-1 in stromal fibroblasts, endothelial cells and epithelial cells in breast cancers. In the endothelial cells, upregulation occurred during the onset of angiogenesis around the *in situ* carcinoma. In ovarian (Davidson *et al*, 2001) and lung carcinomas (Bolon *et al*, 1995), expression of Ets-1 has also been observed in both stromal and tumour cells. In oral squamous cell cancers, Ets-1 was found in endothelial cells of well-vascularised tumours (Pande *et al*, 1999).

In the present study, no significant correlation was found between Ets-1 expression and either tumour size, nodal status, ER or PR status. In colorectal cancer, however, expression of Ets-1 protein was found to be significantly associated with lymph node status, depth of invasion and lymphatic invasion (Nakayama *et al*, 2001). Also, in lung cancer, Ets-1 protein levels correlated with tumour size, lymph node status and tumour stage (Bolon *et al*, 1995).

Although no significant relationship was found between Ets-1 expression and established prognostic factors in this study, the p33, p51 and p52 forms of the Ets-1 protein correlated significantly with uPA levels. uPA is a serine protease causally involved in invasion and metastasis, and is one of the most potent biological prognostic factors so far described in breast cancer (Duffy *et al*, 1998). Recently, its prognostic value was also validated for node-negative breast cancer patients using two different level 1 evidence studies, that is, in both a randomised prospective trial (Foekens *et al*, 2000) and a pooled analysis (Look *et al*, 2002). Consistent with our studies showing a significant relationship between Ets-1 and uPA, Span *et al* (2002) recently reported an association between Ets-1 and poor prognosis in breast cancer. In contrast to our results, Span *et al* (2002) found no significant correlation between Ets-1 and uPA in breast cancer. A significant relationship, however, was found between Ets-1 and PAI-1, an inhibitor of uPA (Span *et al*, 2002). The significant correlation between Ets-1 proteins, that is, p52, p51, p33 and uPA, in this study is consistent with previous reports showing the presence of EBSs in the enhancer/promoter elements of the *uPA* gene (Watabe *et al*, 1998; Sementchenko and Watson, 2000). Indeed, evidence from multiple model systems suggest that the Ets-1 transcription factor may be one of the important regulators controlling the involvement of uPA in the invasive process (Watabe *et al*, 1998; Nakada *et al*, 1999; Sementchenko and Watson, 2000).

In addition to correlating with uPA, we also found a significant relationship between the p52 form of Ets-1 and HER-2/*neu*. HER-2/*neu* is a proto-oncogene overexpressed in 15–30% of invasive breast cancers (Slamon *et al*, 1989). In model systems, overexpression has been associated with both tumorigenesis and metastasis (Slamon and Clark, 1988). In human breast cancers, increased levels are generally associated with adverse outcome (Allred *et al*, 1992). Previously, another member of the Ets family, that is, PEA3, was also found to correlate with HER-2/*neu* in breast cancer (Shepherd *et al*, 2001) and EBSs have been found in the HER-2/*neu* promoter (Chiang *et al*, 2000). Our finding of a correlation between Ets-1 protein levels and both HER-2/*neu* and uPA is consistent with previous data showing a role for Ets-1 in invasive processes (Vandenbunder *et al*, 1994a; Wernert *et al*, 1994; Nakada *et al*, 1999; Pande *et al*, 1999; Kitange *et al*, 2000; Naito *et al*, 2000).

In conclusion, this is one of the most detailed reports to date on an Ets protein in a human cancer. Our results show that certain forms of the Ets-1 protein, that is, p52 and p51, are expressed at much higher levels in breast cancer compared to fibroadenomas. Since multiple signalling pathways converge on Ets transcription factors (Wasylyk *et al*, 1998), the latter should be logical targets for new anticancer agents. Finally, since Ets-1 proteins correlate with both uPA and HER-2/*neu*, high levels are likely to be associated with aggressive disease.
